# The effects of dopamine on digit span in Parkinson’s disease

**DOI:** 10.1186/s40734-016-0033-z

**Published:** 2016-03-07

**Authors:** Clara Warden, Jaclyn Hwang, Anisa Marshall, Michelle Fenesy, Kathleen L. Poston

**Affiliations:** Department of Psychology, Stanford University, Stanford, CA USA; Department of Neurology & Neurological Sciences, Stanford University, Stanford, CA USA; Department of Neuroimaging, King’s College London, London, UK; Department of Psychology, University of California, Los Angeles, CA USA; Department of Neurosurgery, Stanford University, Stanford, CA USA

**Keywords:** Parkinson’s disease, PD, Digit span backward, Digit span forward, Dopamine, Working memory, Cognitive impairment, Dementia

## Abstract

**Background:**

Parkinson’s disease patients are at an elevated risk of developing cognitive impairment. Although cognitive impairment is one of the strongest predictors of quality of life, dopaminergic anti-parkinsonian medications are designed to target motor symptoms. However, there is substantial evidence that dopamine also impacts cognition, in particular working memory. It is therefore critical for movement disorders physicians to understand the potential dopaminergic effects on working memory when prescribing these medications.

Verbal digit span tasks offer a potentially straightforward and quick assessment of baseline working memory. Moreover, Digit Span Backward was recently validated as a screening tool for mild cognitive impairment in Parkinson’s disease when participants were medicated. Research indicates that the interaction between dopamine and working memory follows an Inverted-U shaped curve, but the effect of dopamine on Digit Span has not been well studied.

Our study seeks to: (1) determine the validity of verbal Digit Spans for detecting cognitive impairment in Parkinson’s disease patients both ON and OFF medications; and (2) ascertain the effects of dopaminergic medications on verbal Digit Span.

**Methods:**

We recruited 64 Parkinson’s disease patients and 22 age-and education-matched controls. Parkinson’s patients completed Digit Span Backward and Digit Span Forward ON and OFF medications, while healthy controls completed them once. All participants were categorized by cognitive diagnosis using level-II consensus criteria.

**Results:**

Digit Span Backward successfully identified mild cognitive impairment in Parkinson’s disease, both ON and OFF medications. Combining patients with and without cognitive impairment, we found that dopamine significantly improved performance on Digit Span Backward, but not Forward. In a secondary analysis, we found this dopaminergic improvement was restricted to the Low baseline working memory group; the High baseline working memory group was unaffected.

**Conclusions:**

This study provides evidence for Digit Span Backward as a screening tool for working memory impairment in Parkinson’s disease and for its utility in measuring baseline working memory. Moreover, it reveals a partial beneficial effect of dopamine on Digit Span in Parkinson’s disease patients.

## Background

Parkinson’s disease (PD) patients are at an elevated risk of developing cognitive impairment and dementia [1, 2]. One common, early impairment is a working memory (WM) deficit [3]. WM is the ability to hold and manipulate information in short-term storage for task-relevant purposes; WM is crucial to many higher level cognitive processes such as learning, language comprehension, and reasoning [4, 5]. Although cognitive impairment is one of the strongest predictors of quality of life [6, 7], there are limited treatments targeting PD cognitive symptoms [8]. In the clinic, motor symptoms are treated with dopaminergic medications that alleviate the chronic dopamine depletion that defines PD; however, the effects of these medications on cognition are still poorly understood.

In 1979, Brozoski et al first demonstrated that dopamine depletion from the striatum to the prefrontal cortex in monkeys led to severe impairment on a delayed response task [9]. In the intervening years, there has been growing evidence that dopamine and WM are closely linked [10–17]. Today, research indicates that WM impairment in PD is likely a down-stream effect of nigrostraital dopamine depletion [18]. However, it remains unclear if dopamine depletion is universally detrimental to WM and whether dopamine replacement leads to improvement for all individuals. In a recent review, Cools and D’Esposito argued that poor WM reflects an imbalance between the striatum and the prefrontal cortex, both of which are modulated by dopamine [10, 16, 17, 19–23]. They proposed a double Inverted-U model to describe the relationship between dopamine and WM. This model predicts that dopamine’s effect on WM depends on both individual baseline WM and the specific task being tested [24]. The model holds strong implications for the effects of dopaminergic medications on cognition in PD patients in particular; individuals with intrinsic deficits might benefit from dopamine replacement while those with more superior baseline WM capabilities might suffer. For this reason, it is crucial for clinicians to be able to accurately determine baseline levels of WM in PD patients and predict the effects of dopaminergic medications on WM.

Verbal digit span tasks offer a potentially straightforward and quick assessment of baseline WM. Biundo et al recently validated Digit Span Backward as a diagnostic tool for determining cognitive impairment in PD [25]. However, this study did not consider the possible effects of dopamine since all individuals were tested ON dopaminergic medications. Owing to the strong evidence for a dopaminergic effect on WM, it is pertinent to investigate whether Digit Span remains a valid diagnostic tool OFF medications. Moreover, previous studies that examined the effect of dopamine and PD on Digit Span Backward and Digit Span Forward have reported conflicting findings [11, 26]. It is important to examine the effect of dopaminergic medications on both tasks in a large PD sample.

Our study seeks to: (1) determine the validity of verbal Digit Spans for detecting cognitive impairment in PD patients; and (2) ascertain the effects of dopaminergic medications on verbal Digit Span. In a secondary analysis, we explored the possibility of an Inverted-U effect of dopamine on verbal Digit Span. We tested 64 PD participants ON and OFF medications on Digit Span Forward and Backward.

## Methods

### Subjects

We recruited 64 participants with idiopathic Parkinson’s disease from the Stanford Movement Disorders Clinic and the surrounding community (Table 1). Inclusion criteria were as follows: (1) Age between 45–90 years, (2) fluency in English, (3) right-handed, (4) diagnosis of PD by a board-certified neurologist with specialty training in movement disorders (KLP) based on UK Parkinson’s Disease Society Brain Bank criteria [27], (5) at least two years of a PD diagnosis, (6) at least 20 % improvement in the Movement Disorders Society-United Parkinson’s disease Rating Scale motor score (MDS-UPDRS-III) [28] when ON dopaminergic medications, and (7) no history of other significant neurological disease, serious psychiatric illness, substance abuse, or head trauma.

**Table 1:** Demographics for healthy control and Parkinson’s disease participants

	HC	PD ALL	PD no-MCI	PD-MCI	PDD	*p*
N	22	64	28	22	14	
Male/Female	8/14	35/29	13/15	14/8	8/6	
Age (years)^^^	65.10 ± 6.89	68.36 ± 7.91	65.11 ± 7.21	69.14 ± 7.98	73.21 ± 6.68	* #
Education (years)^^^	16.81 ± 2.03	16.44 ± 2.42	16.48 ± 2.36	16.41 ± 2.56	16.71 ± 2.16	NS
Duration (years)^^^	n/a	5.92 ± 4.18	5.04 ± 3.47	6.64 ± 4.82	6.57 ± 4.38	NS
MDS-UPDRS III (OFF)^^^	n/a	37.47 ± 10.94	35.79 ± 10.53	34.00 ± 9.40	46.79 ± 9.37	# %
MDS-UPDRS III (ON)^^^	n/a	21.54 ± 11.11	19.81 ± 9.62	18.89 ± 10.32	29.85 ± 11.67	# %
LEDD^^^	n/a	695.4 ± 366.1	657.8 ± 382.7	831.8 ± 325.8	556.2 ± 344.1	NS
BDI^^^	3.64 ± 3.88	11.13 ± 8.34	9.26 ± 7.58	11.09 ± 7.08	13.92 ± 11.39	**
BAI^^^	2.68 ± 2.85	11.09 ± 8.80	11.43 ± 9.00	11.32 ± 8.34	10.04 ± 9.63	**

*PD* Parkinson’s disease, *PDD* PD with Dementia, *PD-MCI* PD with mild cognitive impairment, *PD no-MCI* PD with no cognitive impairment, *HC* healthy controls, *MDS-UPDRS III* Movement Disorders Society-Unified Parkinson’s disease Rating Scale, motor scale, *LEDD* levodopa equivalent daily dose, *BDI* Beck’s Depression Inventory, *BAI* Beck’s Anxiety Inventory

^^^ = mean ± standard deviation; * = *p* < .05 PDD vs HC; **#** = *p* < .05 PDD vs PD no-MCI; **%** = *p* < .05 PDD vs PD-MCI; ** = *p* < 0.05 HC vs All PD groups; NS = not significant

In addition, we recruited 22 age- and education-matched healthy controls (HC). Inclusion criteria were as follows: (1) Age between 45–90 years, (2) fluency in English, (3) right handed, (4) no history of significant neurological disease, serious psychiatric illness, substance abuse, or head trauma and (5) no history of cognitive impairment during phone screening.

All participants provided written informed consent to participate in the study following protocols approved by the Stanford Institutional Review Board.

### Clinical evaluation

All PD and HC participants performed a neuropsychological battery that included at least two tests for each of the five cognitive domains (memory, language, executive function, visuospatial, working memory/attention), a Montreal Cognitive Assessment (MoCA), and the Clinical Dementia Rating (See Table 2). PD participants were categorized as PD without cognitive impairment (PD no-MCI), PD with mild cognitive impairment (PD-MCI), or PD with dementia (PDD) according to published criteria [27, 29]. PD-MCI was defined as exceeding 1.5 standard deviations below age- and education-matched normative values on two tests, either in the same domain or separate ones [27]. A designation of dementia was reserved for those individuals who received a score of greater than or equal to 1 on the CDR [29]; all dementia patients were independently categorized as impaired on multiple domains [30]. As recommended by current criteria [27], the comprehensive neuropsychological testing was performed ON medications to minimize motoric interference in testing.

**Table 2:** Neuropsychological battery

	HC	PD no-MCI	PD MCI	PDD	*p*-valueHC vs PD no-MCI
MoCA	27.52 ± 1.97	27.57 ± 2.01	23.50 ± 2.72	16.79 ± 1.97	0.93
DRS	140.19 ± 2.99	139.43 ± 2.81	135.45 ± 5.60	118.43 ± 16.01	0.37
CVLT LD Free	12.19 ± 2.71	11.07 ± 3.1	5.82 ± 2.75	3.29 ± 3.87	0.19
BVMT-R	10.1 ± 2.45	10.25 ± 1.92	5.64 ± 2.66	1.92 ± 2.47	0.80
JLO	26.24 ± 3.79	25.04 ± 3.39	21.14 ± 5.16	17.73 ± 5.85	0.25
HVOT	26.71 ± 1.91	25.88 ± 2.38	22.14 ± 4.28	17.69 ± 6.72	0.19
SDMT Oral	57.14 ± 9.46	56.46 ± 13.09	40.32 ± 11.98	17.75 ± 9.02	0.84
COWAT	46.43 ± 12.29	49 ± 12.96	33.77 ± 18.14	22.43 ± 10.06	0.49
WAIS-IV Digit total	17.43 ± 3.34	18.93 ± 3.89	15.82 ± 4.86	11.43 ± 2.24	0.16
Trails B	67.62 ± 23.44	68.39 ± 26.12	161.41 ± 91.46	280.5 ± 48.82	0.92
Stroop	-2.16 ± 14.76	-3.29 ± 7.57	-7.55 ± 9.55	-12.07 ± 5.63	0.73
BNT	29.14 ± 0.91	27.82 ± 3.94	24.64 ± 4.96	25.43 ± 2.93	0.14
DKEFs Verbal	20.9 ±5. 12	22.32 ± 6.34	17.09 ± 6.22	10 ± 5.39	0.41

Table depicts the mean ± standard deviation for the demographic information and neuropsychological test data, with p-values derived from independent-sample t-test between HC and PD no-MCI

*PD* Parkinson’s disease, *HC* Healthy control, *MoCA* Montreal Cognitive Assessment, *CVLT LD Free* California Verbal Learning Test, Long Delay Free Recall, *BVMT-R* The Brief Visuospatial Memory Test-Revised, *JLO* Judgment of Line Orientation, *HVOT* Hooper Visual Organization Test, *SDMT Oral* Symbol Digit Modalities Test, oral, *COWAT* Controlled Oral Word Association Test, *WAIS-IV Digit total* Wechsler Adult Intelligence Scale, Digit combined total, *Trails B* Trail Making Test B, *Stroop* Golden version of Stroop test, Interference score, *BNT* Boston Naming Test, *DKEFs Verbal* Delis-Kaplan Executive Function System, Verbal score

In order to determine the effect of dopamine on Verbal Digit Span, PD participants performed the WAIS-IV Digit Span Backward and Digit Span Forward twice, once in the OFF and once in the ON state, counterbalanced, and with a least a two week interval period. The Digit Span OFF and ON was performed on the same day as the MDS-UPDRS-III OFF and ON, respectively, to control for potential motor fluctuations. The Neuropsychological battery was performed in the ON medication state, as recommended, and on a separate day [31]. Critically, we administered the oral version of the Digit Span to minimize potential bias from bradykinesia or dyskinesias.

In PD participants, the OFF state was defined as ≥ 72 h off extended release dopamine agonists, selective MAO-B inhibitor, and long-acting levodopa, and ≥ 12 h off short acting dopamine agonists and levodopa. The ON state was defined as the patients taking their normal daily medications in the optimally medicated state, as determined by both the patient and the movement disorders neurologist. We took steps to minimize the influence of motor fluctuations on the tests performed ON medications. First, the researchers documented the patient’s last dose of medications, and the next scheduled dose, to confirm the testing was during the optimal time in relation to scheduled medications. Second, it was documented that the patient remained in the ON state throughout the 30 min of the MDS-UPDRS-III and Digit Span. With regards to dopamine replacement therapy, 41 participants were taking levodopa and a dopamine agonist, 2 were taking levodopa and a MAO-B inhibitor, 18 were taking levodopa, a dopamine agonist, and a MAO-B inhibitor, 1 was taking a dopamine agonist and a MAO-B inhibitor, 1 was taking only an MAO-B inhibitor, and 1 was taking a combination of levodopa, a dopamine agonist, a MAO-B inhibitor and a COMT inhibitor.

### Statistical analysis

All statistical analysis were conducting using IBM SPSS Statistics version 22.0 [32].

## Results

### Demographics

HC participants were age-matched with the overall PD group and with the PD no-MCI and PD-MCI groups.) HC and PD no-MCI groups were significantly younger than the PDD group. There were no significant differences in education, duration of disease, levodopa equivalent doses (LEDD), depression, or anxiety across all PD groups (Table 1). In addition, there were no significant differences between the HC and PD no-MCI groups on any of the neuropsychological tests administered (Table 2). 59 participants completed the digit span ON and OFF dopaminergic medications, 1 completed it only OFF medications, and 4 completed it only ON medications. Only participants with both an ON and an OFF session were included in the analysis of medication effects. 34 participants were tested ON medications first while 25 were tested OFF medications first. Repeat measure ANOVAs with repeated factor Medications (ON, OFF) and between-subjects factors Session (ON first, OFF first) and Baseline WM (High, Low) did not reveal any session effects.

### Between group analysis

**Fig. 1:**
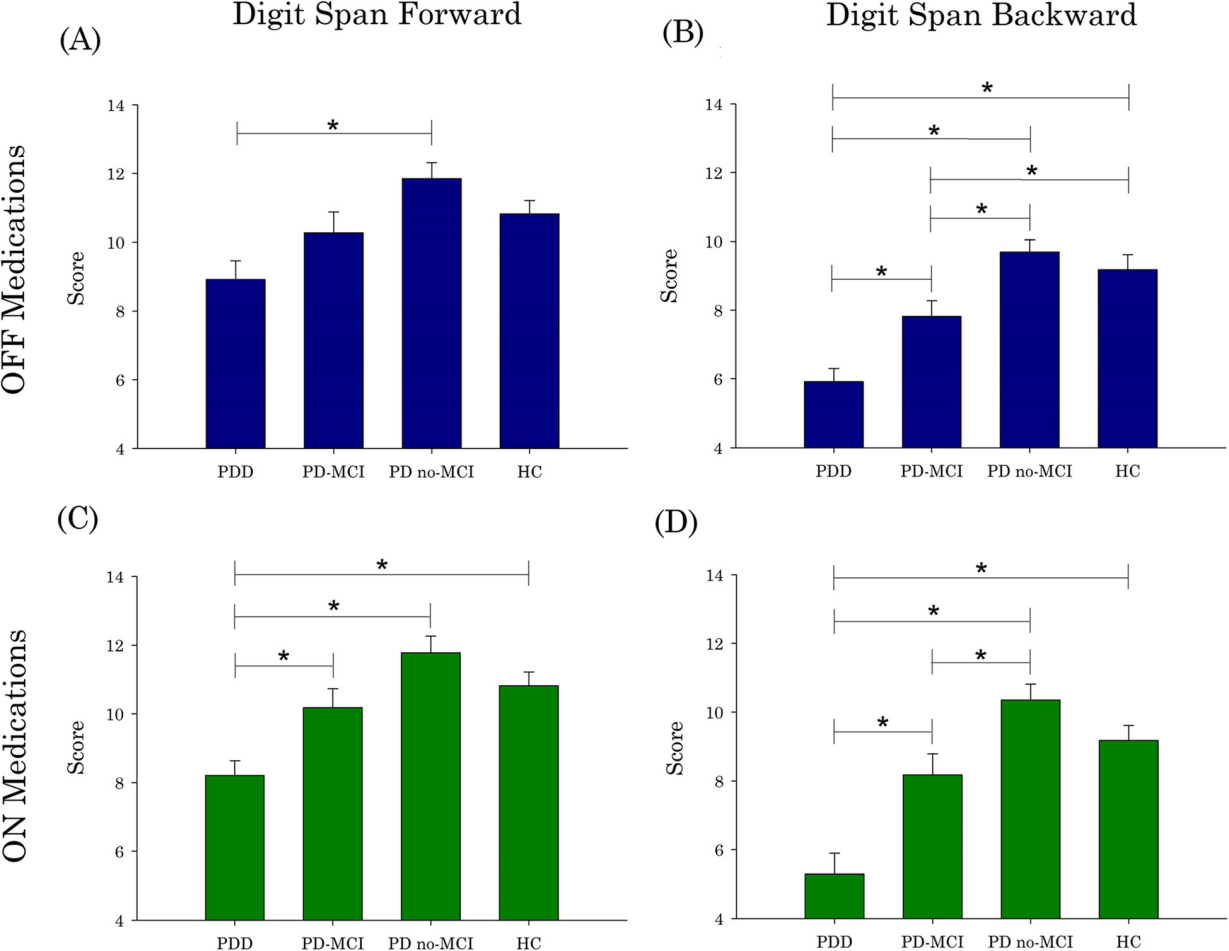
Performance (mean ± SE) on Digit Span Backward and Forward. The effect of Group (PDD, PD-MCI, PD no-MCI, HC) was significant in all cases. Digit Span Forward only distinguished PDD (**a** and **c**). However, Digit Span Backward, both ON and OFF medications, successfully identified PD-MCI (**b** and **d**). Performance represents total scores as tabulated using WAIS-IV guidelines, not digit span capacity. PD = Parkinson’s disease; PDD = PD with dementia; PD-MCI = PD with mild cognitive impairment; PD no-MCI = PD with no cognitive impairment; HC = healthy controls. * = *p* < .05

Due to the non-normal distribution of the PDD group, Kruskal-Wallis tests with between-subjects factor Group (HC, PD no-MCI, PD-MCI, PDD) and post-hoc stepwise step-down procedures were used to analyze the differences in Digit Span Forward and Backward scores between clinical groups (Fig. 1). Four separate Kruskal-Wallis tests were conducted: Digit Span Forward with PD OFF, Digit Span Forward with PD ON, Digit Span Backward with PD OFF, and Digit Span Backward with PD ON. Moreover, we conducted a Kruskal-Wallis test with between-subject factor Group (HC, PD no-MCI, PD-MCI, PDD) for the abbreviated digit span on the MoCA.

#### Digit span forward, PD OFF

The effect of Group was significant (*p* < .005). Post-hoc analysis revealed that PDD performed significantly worse than PD no-MCI (*p* < .05). No other groups were significantly different (Fig. 1a).

#### Digit span forward, PD ON

The effect of Group was significant (*p* < .001). Post-hoc analysis revealed that PDD performed significantly worse than PD no-MCI, PD-MCI, and HC (*p* < .05 in all cases). No other groups were significantly different (Fig. 1c).

#### Digit span backward, PD OFF

The effect of Group was significant (*p* < .001). Post-hoc analysis revealed that PDD performed significantly worse than HC, PD no-MCI, and PD-MCI (*p* < .05 in all cases). Moreover, the PD-MCI group performed significantly worse than PD no-MCI and HC (both *p* < .05). PD no-MCI was not significantly different from HC (Fig. 1b).

#### Digit span backward, PD ON

The effect of Group was significant (*p* < .001). Post-hoc analysis revealed that PDD performed significantly worse than HC, PD no-MCI, and PD-MCI (*p* < .05 in all cases). PD-MCI also performed significantly worse than PD no-MCI (*p* < .05). PD no-MCI and PD-MCI were not significantly different from HC (Fig. 1d).

#### MoCA digit span sub-score

The effect of Group was not significant. The majority of individuals, from all groups, achieved a perfect score of 2/2: 78.6 % of PDD, 90.9 % of PD-MCI, 89.3 % of PD no-MCI and 100 % of HC.

### Effect of dopaminergic medication

**Fig. 2:**
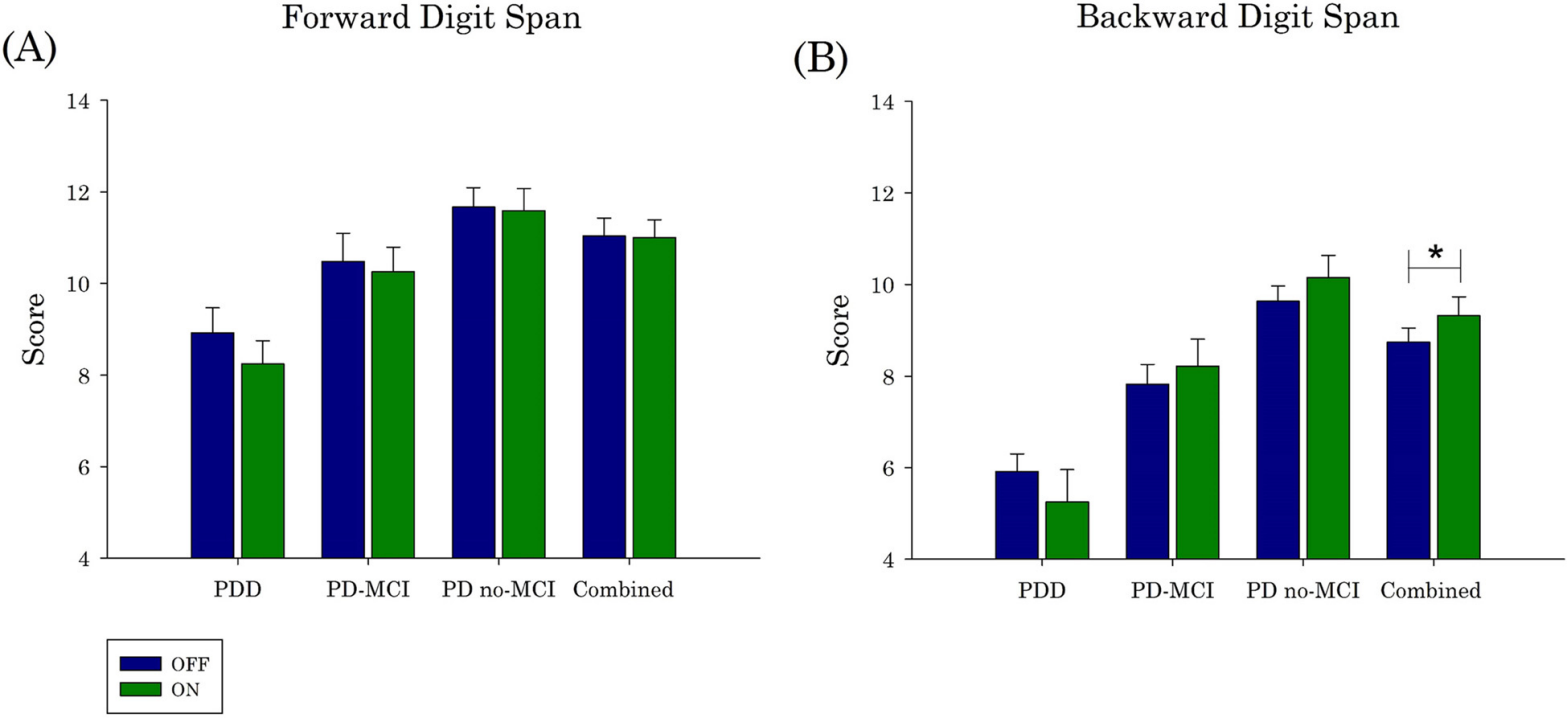
The effect of dopaminergic medication on performance on Digit Span Backward and Forward. There was no significant effect of dopaminergic medications on Digit Span Forward (**a**). However, a significant effect of medications was detected in Digit Span Backward. The Combined PD group (PD-MCI and PD no-MCI) performed significantly better ON medications than OFF medications (**b**). Error bars represent the Standard Error of the mean. PD = Parkinson’s disease; PDD = PD with dementia; PD-MCI = PD with Mild Cognitive Impairment; PD no-MCI = PD with no cognitive impairment. * = *p* < .05

In order to isolate the effect of dopaminergic medications on WM, as measured by Digit Spans, we compared performance within groups ON and OFF medications. For PD-MCI and PD no-MCI groups we conducted paired sample Student’s T-tests between ON and OFF sessions. For the PDD group we conducted the Related-Samples Wilcoxon Signed Rank Test. There was no significant effect of dopaminergic medications in Digit Span Backward or Forward in PD no-MCI, PD-MCI, or PDD (Fig. 2a). However, when PD no-MCI and PD-MCI were combined, a significant effect of the medications was detected. In Digit Span Backward, PD participants performed significantly better ON compared to OFF dopaminergic medications (*p* = .043) (Fig. 2b).

### Effect of baseline WM

Prior PD studies have shown that WM can have an Inverted-U response to dopamine, where patients with poor baseline (OFF dopamine) performance show improvement after dopamine replacement and those with good baseline performance show worsening [21, 22, 24, 33]. Therefore, we conducted a secondary analysis to explore a possible interaction between baseline WM and the effect of dopaminergic medications on Digit Span performance in our sample. We only included PD no-MCI and PD-MCI in this analysis due to the non-normal distribution of the PDD group and to eliminate the possible confounding factors of mixed pathology, elevated age, and severe impairment in alternate cognitive domains that could impact performance (e.g. severe episodic memory or executive impairment that prohibits encoding). As the cross-sectional nature of our study prohibited evaluating premorbid WM performance, we followed the protocol of previous studies and used the median of the combined group Digit Span Backward OFF to determine the cut-off score differentiating High versus Low baseline WM in our cohort [33]. Digit Span Backward ≥ 9 categorized an individual as High WM while < 9 was categorized as Low WM. Of the PD-MCI group, 16 were determined to have Low baseline WM and 6 to have High baseline WM. Of the PD no-MCI group, 9 fell into the Low baseline WM group and 19 in the High baseline WM group. There were no significant differences between motor, depression, or anxiety scores between the High and Low groups (Table 3). We conducted separate analysis of Digit Span Forward and Backward, each using a mixed measures ANOVA with between-subjects factor Group (High Baseline WM, Low Baseline WM) and within-subjects factor Medication (ON, OFF).

**Table 3:** High versus low baseline working memory

	Low Baseline Working Memory	High Baseline Working Memory	*p*
N	25	25	
PD no-MCI/PD-MCI	9/16	19/6	
Age (years)	65.36 ± 7.26	68.72 ± 8.07	NS
Education (years)	16.16 ± 2.90	16.56 ± 2.08	NS
Duration (years)	6.17 ± 5.02	5.20 ± 3.18	NS
MDS-UPDRS III (OFF)	33.32 ± 9.22	36.68 ± 9.89	NS
MDS-UPDRS III (ON)	19.83 ± 9.22	18.75 ± 10.76	NS
BDI	11.13 ± 6.84	9.80 ± 7.99	NS
BAI	11.72 ± 10.54	11.04 ± 6.38	NS

Table depicts the mean ± standard deviation for the demographics of the High and Low Baseline Working Memory groups

*PD* Parkinson’s disease, *PD-MCI* PD with mild cognitive impairment, *PD no-MCI* PD with no cognitive impairment, *MDS-UPDRS III* Movement Disorders Society-Unified Parkinson’s disease Rating Scale, motor scale, *BDI* Beck’s Depression Inventory, *BAI* Beck’s Anxiety Inventory, *NS* not significant between groups on t-test

#### Digit span forward

**Fig. 3:**
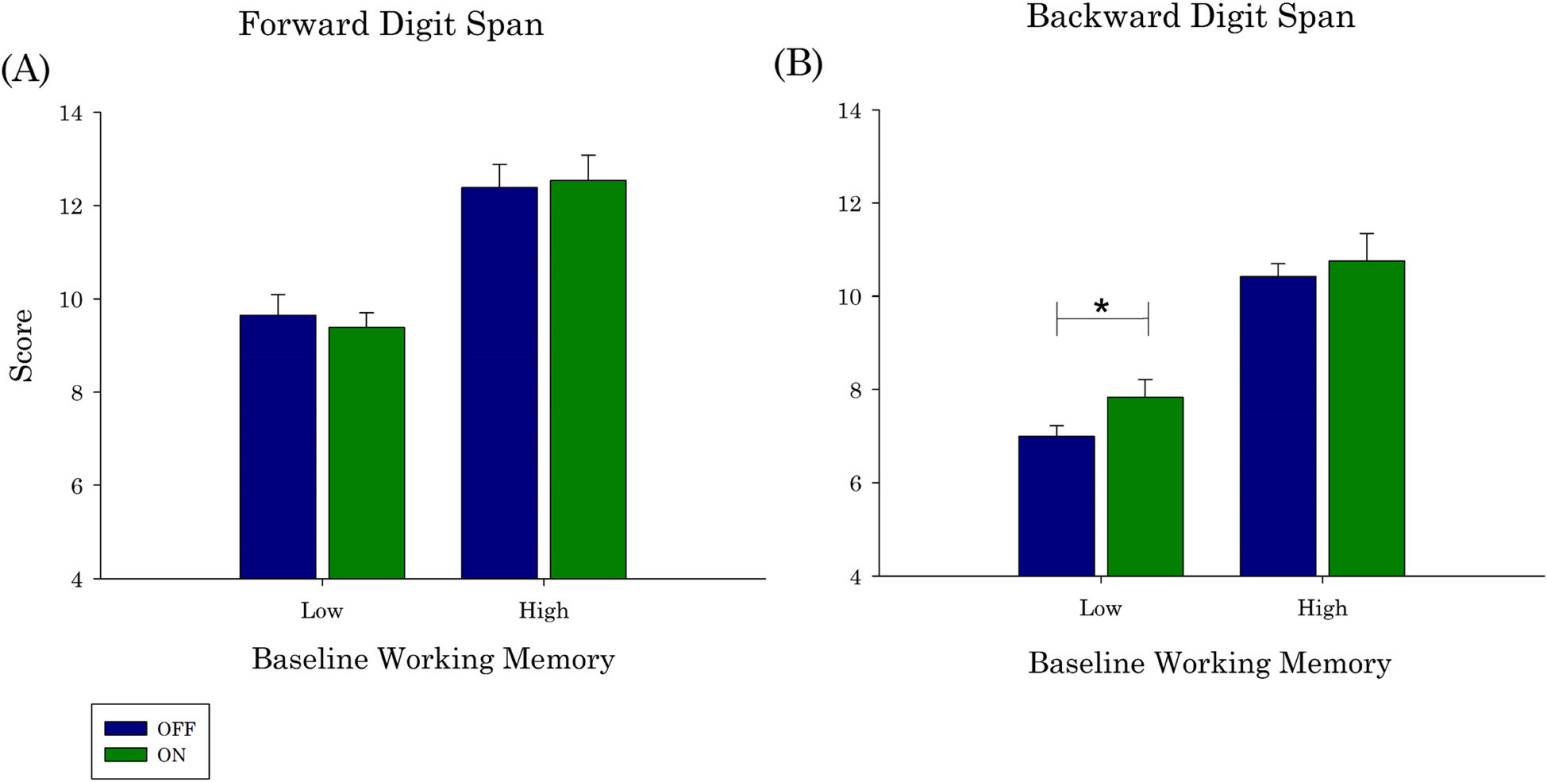
Dopaminergic effect is dependent on baseline WM. We determined the interaction between baseline WM and dopamine using Digit Span. No significant effect of dopamine was detected in the Digit Span Forward in either the Low or High baseline WM groups (**a**). However, in Digit Span Backward, the Low baseline WM group significantly improved ON medications. The High baseline WM Group was unaffected (**b**). Error bars represent the Standard Error of the mean. PD = Parkinson’s disease; PD-MCI = PD with mild cognitive impairment; PD no-MCI = PD with no cognitive impairment; WM = working memory. * = *p* < .05

There was a main effect of Group (*p* < .001), but no main effect of Medications. There was no interaction effect (Fig. 3a).

#### Digit span backward

There was a main effect of Group (*p* < .001), a main effect of Medication (*p* = .042), and no interaction effect; however post-hoc Student’s T-tests revealed the Medication effect was only in the Low WM group (*p* = .002). Indeed, the Low WM group performed significantly better ON compared to OFF dopaminergic medications (Fig. 3b).

## Discussion

We investigated the utility of Digit Span Backward and Digit Span Forward as tools for identifying cognitive impairment in PD and the effects of dopaminergic medications on Digit Span in PD. We found that Digit Span Backward best distinguished cognitive classification (no-MCI, MCI and dementia) in our cohort. Moreover, exploratory analysis revealed a partial beneficial effect of dopaminergic medications on Digit Span Backward, with low WM individuals improving on dopamine and high WM individuals demonstrating no benefit. Our research provides evidence for Digit Span Backward as a screening tool for WM impairment in PD and for its utility in measuring baseline WM.

### Digit span backwards in PD

Our findings corroborate those of Biundo et al., who reported that Digit Span Backward was one of five cognitive assessments that reached diagnostic and screening validity for PD-MCI, while Digit Span Forward failed to do so [25]. While this study only tested participants ON dopaminergic medications, our study provides further evidence that Digit Span Backward successfully distinguishes between PD no-MCI, PD-MCI, and PDD regardless of whether performed ON or OFF medications. By contrast, we found Digit Span Forward was only abnormal in PDD.

Moreover, we found Digit Span Backward offers greater insight into WM impairment than the limited digit span conducted in the MoCA. Currently, the MoCA is among the most commonly used tools for screening PD cognitive impairment in the clinical setting [34]. However, our findings indicate the limited digit span in the MoCA is a very weak detector of WM impairment, especially among a population of highly educated individuals. This is in accordance with the finding that education is a strong predictor of verbal Digit Span, with higher educational attainment leading to greater span capacity [35]. Years of education in our cohort averaged 16 and the limited digit span on the MoCA failed to differentiate between any of the cognitive groups; even the vast majority of participants with dementia achieved a perfect score. Thus, we suggest Digit Span Backward could be a relatively simple compliment to the MoCA and could be used in clinic to screen highly educated PD patients who are suspected of a WM deficit.

### Dopaminergic effect on digit span

Prior studies in PD have reported conflicting effects of dopaminergic medications on the verbal Digit Span, likely due to small sample sizes. Zokaei et al previously reported a significant dopaminergic improvement in WM in PD patients as detected by a WM precision task, but indicated this improvement was not detected in Digit Span Backward or Forward [26]. On the other hand, Cools et al found PD patients OFF medications performed worse than healthy controls on Digit Span Backward, but this impairment was rectified by dopaminergic medications [11]. Our study found a modest degree of dopaminergic improvement similar to that of Cools et al, but validates their finding in a much larger cohort. In addition, our study expands upon Cools et al by including a full neuropsychological assessment to confirm that the dopaminergic improvement is seen in both cognitively impaired and cognitively normal PD patients, depending on the baseline working memory.

An Inverted-U effect of dopaminergic medications has previously been described in other WM tasks, such as delay-response [36], and attentional set shifting [23, 33, 37]. We conducted a secondary analysis to determine a possible interaction between baseline WM and the effect of dopaminergic medications specifically on the Digit Span. In accordance with other studies exploring the Inverted-U model [21, 24], we only detected a dopaminergic improvement when groups were divided by baseline WM (Low versus High) and not by global cognitive classification (MCI versus no-MCI). This is likely because we our Low and High WM groups included a distribution of both PD no-MCI and PD-MCI individuals; 9 out of 28 PD no-MCI had low baseline WM and 16 out of 22 PD-MCI had low baseline WM. While it may seem counterintuitive that patients categorized as no-MCI have low WM, this is likely due to the threshold for determining PD no-MCI (1.5 SD below age- and education- normative values in at least two out of 10 tests), which allows patients to have one test more than 1.5 SD below normative values, or have sub-threshold poor performance on multiple tests. By contrast, PD patients who are globally categorized as cognitively impaired do not necessarily have poor WM, as they could have visuospatial or episodic memory impairments. Thus, our study highlights the limitations of using global cognitive categorization when examining a dopaminergic effect on a specific cognitive ability and the value of identifying validated tests for PD patients in individual cognitive domains. Due to the cross-sectional nature of our study, baseline WM was determined using performance on Digit Span Backward OFF medications. Although pre-morbid WM would have been a preferable baseline, using performance when OFF medications enabled us to isolate the effect of dopamine from that of the primary disease.

The Inverted-U hypothesis predicts that the effect of dopamine depends upon baseline WM and the nature of the cognitive task being performed. Specifically, tasks that require high levels of stabilization should benefit from high levels of dopamine in the prefrontal cortex relative to the striatum [10, 22, 24, 38]. Therefore, poor baseline WM suggests an imbalance of dopamine between the prefrontal cortex and striatum, and high baseline WM indicates an optimized balance [22, 24, 38]. In addition, this hypothesis suggests that low WM individuals should benefit from dopaminergic medications on straightforward WM tasks that require cognitive stabilization, such as Digit Span, while high WM individuals should be detrimentally affected. Our study was not optimized to fully explore this relationship because we could only divide our cohort into two groups (high and low WM) and a true Inverted-U effect would ideally show those who improve, those who do not change, and those who are detrimentally affected; however some of our findings can be explained within this framework. For instance, dopaminergic medications might have benefited the low WM individuals by shifting the dopamine balance between the prefrontal cortex and the striatum to a more optimal level [24, 38]. Our high WM individuals were unaffected by dopamine, possibly because dopaminergic medications did not alter the balance (i.e. they were already optimized). An alternative explanation is that, unlike other WM tasks, Digit Span Backward does not follow an Inverted-U dopaminergic response due to the simplicity of the task. Further studies optimized to explore this question, ideally with more difficult WM tasks, could clarify this distinction.

### Practical applications for the digit span backward

As described above, we established Digit Span Backward as an accurate predictor of baseline WM. In concordance with Lewis et al, which revealed effects of dopaminergic medications on the manipulation but not maintenance stage of WM, the Inverted-U was only detected in Digit Span Backward and not Digit Span Forward [15]. Our findings indicate that Digit Span Backward can be used to differentiate between low and high WM individuals in clinical and research settings.

An important consequence of the detected dopaminergic effect on the Digit Span Backward is that the task is a more sensitive determinate of baseline WM when it is conducted OFF medications. As the medications improve performance in the Low WM group but do not affect the High WM group, they effectively blur the line between high and low WM individuals such that in the ON medications state some low WM individuals could inadvertently be categorized as high WM. As such, we recommend the Digit Span Backward be conducted OFF medications if possible when being used to primarily identify low baseline WM individuals.

### Methodology and limitations

Our study has several limitations. It should be noted that our PDD group was significantly older than our PD no-MCI and HC groups. The age difference, in addition to possible mixed-pathology, confounds our ability to determine whether the significantly impaired performance of the PDD group on Digit Span Forward was a direct effect of PDD or influenced by the effect of age. We were also unable to determine baseline WM for the PD patients prior to onset of disease. This limits our ability to determine whether impairments represent an individual’s pre-morbid cognitive ability or are a result of PD. However, our findings remain significant for clinical purposes as they offer clinicians a tool to predict the effects of dopaminergic medications on WM. Moreover, it is difficult to generalize our findings to all PD patients since our cohort was not necessarily representative. Notably, our PD no-MCI and PD-MCI groups did not perform significantly differently than HC on any task, regardless of medication status. This could be due to motivation of PD patients to perform well [39–41], or because 6 out of 22 PD MCI participants had high baseline WM. Generalizability of our findings is also a concern; PD patients were primarily recruited from an academic tertiary care center, resulting in a highly educated cohort.

Finally, we recognize that Digit Span Backward does not test all aspects of WM and cognition. Notably, Cools et al previously reported that improvement in WM on Digit Span Backward was counterbalanced by increased distractibility in PD patients, indicating dopaminergic medications were beneficial to some aspects of cognition and detrimental to others [11]. Further studies should investigate the longitudinal validity of Digit Span Backward and the effects of dopaminergic medications on other cognitive functions, such as attentional control. Moreover, neuroimaging studies can lead to a deeper understanding of whether the dopaminergic effect we detected represents a stabilization of the balance between the prefrontal cortex and the striatum [24].

## Conclusions

Our research supports the use of Digit Span Backward to screen for mild cognitive impairment, particularly among highly educated PD patients, and to detect baseline WM. It also suggests a partial beneficial effect of dopaminergic medications on verbal Digit Span Backward in PD.

## Abbreviations

ANOVA: analysis of variance
BNT: Boston naming test
BVMT-R: the brief visuospatial memory test-revised
COMT: Catechol-O-methyl transferase
COWAT: controlled oral word association test
CVLT LD Free: California verbal learning test long delay free recall
DKEFs Verbal: Delis-Kaplan executive function system, verbal score
HC: healthy controls
HVOT: Hooper visual organization test
JLO: judgment of line orientation
MAO-B: Monoamine oxidase B
MoCA: Montreal cognitive assessment
PD: Parkinson’s disease
PD no-MCI: Parkinson’s disease without cognitive impairment
PDD: Parkinson’s disease with dementia
PD-MCI: Parkinson’s disease with mild cognitive impairment
SDMT Oral: symbol digit modalities test, oral
Stroop: golden version of Stroop test, Interference score
Trails B: trail making test B
WAIS-IV Digit total: Wechsler adult intelligence scale, digit combined total
WM: working memory
